# Diverse Approaches in Wet-Spun Alginate Filament Production from the Textile Industry Perspective: From Process Optimization to Composite Filament Production

**DOI:** 10.3390/polym16131817

**Published:** 2024-06-27

**Authors:** Cansu Var, Sema Palamutcu

**Affiliations:** Department of Textile Engineering, Engineering Faculty, Pamukkale University, 20160 Denizli, Türkiye; spalamut@pau.edu.tr

**Keywords:** alginate filament, wet spinning, textile

## Abstract

Alginate, categorized as a natural-based biodegradable polymer, stands out for its inherently exclusive properties. Although this unique polymer is widely processed using film, coating, and membrane technologies for different usage areas, textile applications are still limited. This study aims to compile promising approaches that will pave the way for the use of wet-spun alginate filaments in textile applications. In this regard, this study provides information about the molecular structure of alginate, the gel formation mechanism, and cross-linking using different techniques. Our literature review categorizes parameters affecting the mechanical properties of wet-spun alginate filaments, such as the effect of ion source and spinning dope concentration, needle diameter, temperature, and coagulants. Following this, a detailed and comprehensive literature review of the various approaches, such as use of additives, preparation of blended filaments, and grafted nanocrystal addition, developed by researchers to produce composite alginate filaments is presented. Additionally, studies concerning the use of different cations in the coagulation phase are reported. Moreover, studies about the functionalism of wet-spun alginate filaments have been offered.

## 1. Introduction

**Figure 1:**
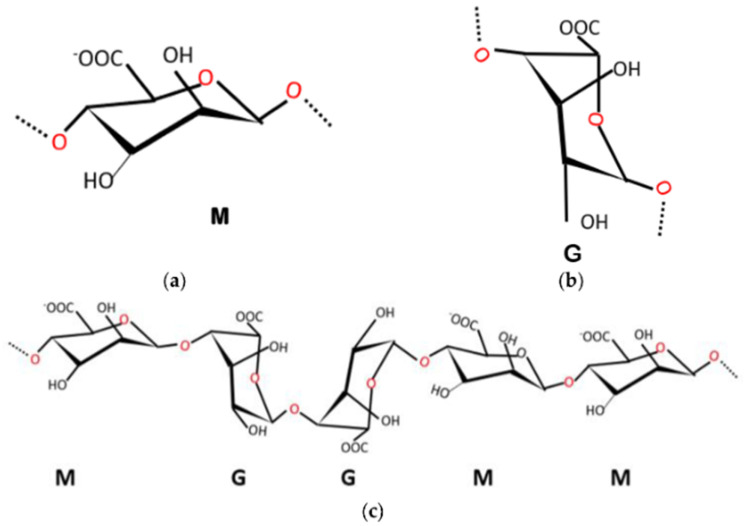
Alginate structure: (**a**) β−D−mannuronic acid (**b**) α−L−guluronic acid, and (**c**) M and G block conformation of alginate [5].

Natural-based biodegradable polymers, which are environmentally friendly alternatives to conventional textile raw materials, are expected to exhibit an important role during the evolution of sustainable and innovative transition of textiles. Among these polymers, alginate is a polysaccharide-based polymer, which is rich in carboxyl groups (−COOH) in its structure. “Alginate” refers to the salts of alginic acid, but it is also utilized to describe all derivatives of alginic acid and alginic acid itself. However, the well-known, main commercial form of alginate is the sodium alginate [1,2]. Commercially available alginate is extracted from several species of algae such as *Laminaria Macrocystis*, *Sargassum*, *Ascophyllum*, *Lessonia*, *Eclonia*, and *Durvillea* [3,4]. Notwithstanding a significant portion of alginate yield from wild seaweed globally, aquaculture as a source of alginate is already utilized in special farms in China [5]. Apart from seaweed, it may be possible to extract alginate from certain bacterial species such as *Azotobacter* spp. and *Pseudomonas* spp. [5,6,7]. The molecular chain of alginate consists of α−L−guluronic acid (G) and β−D−mannuronic acid (M) units, which are arranged to form G blocks (GGGGG), M blocks (MMMMM), or MG (GMGMGM) blocks (Figure 1) [3,5,8,9]. The stereochemical structures of M−G, G−G, G−M, and M−M blocks are illustrated in Figure 1c. The sequence of G and M blocks may vary depending on the type of alginate source [10].

**Figure 2:**
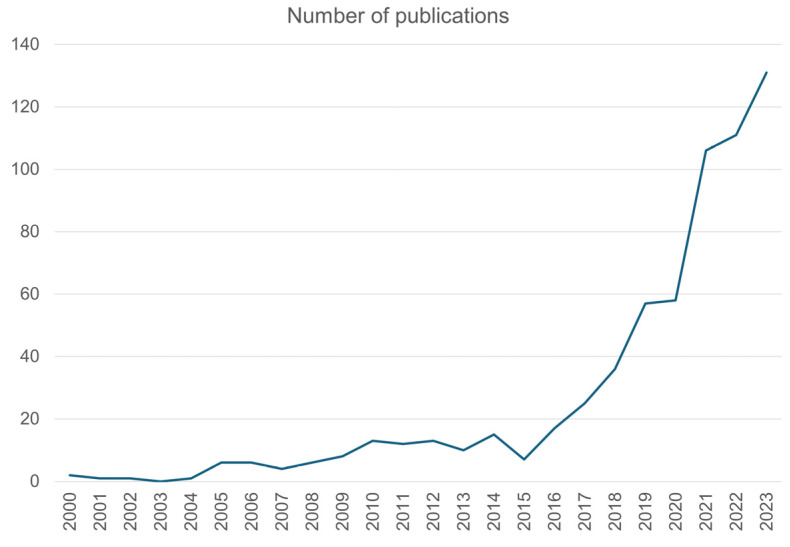
The trend chart of the publications on wet-spun alginate filaments by year, adapted from [27].

This unique polymer stands out with its versatile usage areas thanks to its inherent distinguished properties such as low toxicity and immunogenicity, high hydrophilicity, rapid biodegradation, low cost, good water barrier properties, gel-forming ability, and swelling properties. Alginate has been utilized in different areas including biomedicine applications and tissue engineering, food, pharmaceuticals, drug delivery systems, and traditional wound dressings. Wound closure, burn treatment, hemostasis, hernia, bone and cartilage defect repair, dental impression, and guided bone regeneration are among the specific medical applications of alginate [11,12]. Aside from medical applications, special-use fields for alginate, such as self-healing/rejuvenation of asphalt [13,14,15], wastewater treatment [16], and viscosifiers in textile printing [17], have attracted significant attention from researchers in recent years. Furthermore, the processing of alginate-included leatherette is another important rising research area in the literature [18]. To address the abovementioned use areas, alginate is transformed into many different forms including coating [19], films [20,21,22], membranes [23,24,25,26], and polymer capsules. On the other hand, alginate must be converted to filament form by filament manufacturing technologies including melt spinning or wet spinning to address textile applications. Both for the traditional textile industry and for technical textile applications, its usage area is still on a limited scale and confined to medical textiles. However, as seen in Figure 1, it should be emphasized that scientific studies containing wet spinning of alginate filaments have dramatically increased since 2015. Figure 2 shows that studies on the subject have tended to increase in recent years. It is possible to comment that this increasing trend is parallel to the increasing search for alternative raw materials of the textile industry under the influence of various factors such as environmental sustainability awareness, depletion of resources, economic factors, consumer demands, and regulations.

Specifically, the gel-forming ability of alginate is of paramount importance for manufacturing filaments for textile applications. This distinguished characteristic allows alginate to participate in ionic cross-linking with metal ions such as calcium (Ca^2+^), transforming flexible and durable hydrogels. The gelation properties of alginate largely depend on the molecular weight and the composition of the M, G and MG blocks. In the literature, it is reported that the molecular weight of alginate varies between 32,000 and 400,000 g/mol. Additionally, as the molecular weight increases, the viscous formation ability increases during the gel formation process, which can bring about filament formation control and reduced breaking during the process [10].

## 2. Gel Forming Mechanism of Alginate

**Figure 3:**
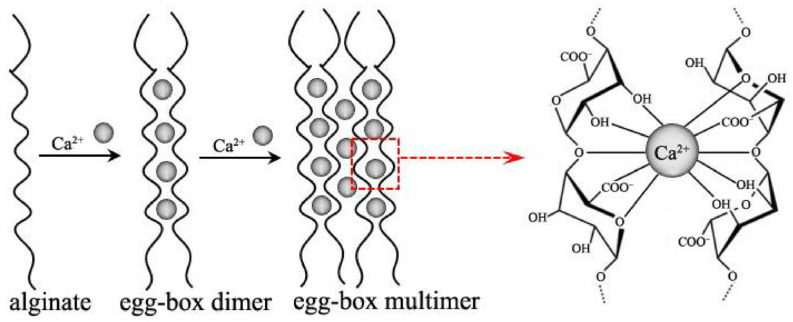
Representative illustration of the egg-box junctions of Ca^2+^ within G blocks [30].

The gelation mechanism of alginate is a complex process and is generally explained in the literature with the “egg-box” structural model (Figure 3). The egg-box model describes the interaction between alginate polymer chains and multivalent cations. In aqueous media, carboxyl groups (–COOH) in alginate structure are transformed into carboxylate ions (–COO). Divalent or trivalent cations replace sodium in alginate, which initiates ionic cross-linking between these metal ions and carboxyl groups and activates the gelation mechanism. Cross-linking typically occurs in the presence of divalent and trivalent metal cations such as Cu^2+^, Ba^2+^, Sr^2+^, Ca^2+^, Mg^2+^, Sr^2+^, Zn^2+^, Mn^2+^, Fe^3+^, Al^3+^, Cr^3+^ [28,29,30,31].

The cross-linking process and the physical and mechanical properties of the resulting gel depend on various factors such as the molecular structure of alginate, the type and concentration of utilized cation, and the gelation duration [29,32]. In many studies, it has been stated that G blocks in the molecular structure participate in cross-linking due to their higher affinity for cation [28,33,34,35,36]. M blocks are widely considered to have a negligible affinity towards cations compared to G blocks, ascribed to their low stereospecificity. Contrary to the majority of the studies stating that G blocks participate in cross-linking during wet spinning of alginate filaments, an approach to researching alginate filaments composed of M blocks was developed by Aneem et al. [37]. On the other hand, Raus et al. [10] and Sahoo and Biswal [4] stated that alginate with high M content tends to be more immunogenic than alginate with high G content. Depending on the M/G ratio, the mechanical characteristics of alginate are greatly affected [28]. Augst et al. [28] stated that G blocks play an important role in the stiffness of the alginate hydrogel, while Cattelan et al. [38] stated that the elastic modulus of alginate hydrogels depends on the content of both G and M blocks. According to Aneem et al. [37], alginate structures rich in M blocks possess enhanced elasticity.

In their study, Kaklamani et al. [29] obtained the highest to lowest elasticity value and gelation rate in Ca^2+^, Sr^2+^ and Mg^2+^ cations, respectively. However, it should be noted that the affinity of each cation towards alginate is at distinct levels. Aneem et al. [37] listed the affinity hierarchy of different cations towards alginate as follows: Pb > Cu > Cd > Ba > Sr > Ca > Co, Ni, Zn > Mn. According to Hu et al. [39], the gel stability decreases in the following order: Ba^2+^ > Cd^2+^ > Cu^2+^ > Sr^2+^ > Ni^2+^ > Ca^2+^ > Zn^2+^ > Co^2+^ > Mn^2+^ > Mg^2+^. Among these cations, Ca^2+^-induced interactions have been the most extensively studied and have consistently attracted the attention of researchers [37,40].

The unique gel-forming mechanism of alginate makes it possible to process it in wet spinning technology. The egg-box model is of critical importance because it is a guiding model in understanding the effect of different ionic interactions on the properties of the resulting filaments and in the design and optimization of textile materials. Some cations used in the gel formation mechanism of alginate and their properties are given below.

### 2.1. Cross-Linking with Divalent Cations

#### 2.1.1. Calcium, Ca^2+^

The most studied divalent cation-inducing gelation of alginate is Ca^2+^, as mentioned above. Ca–alginate gels are mostly formed by extrusion of the alginate solution in a CaCl_2_ bath by the exogenous method. However, the gel that is formed during the cross-linking process with Ca^2+^ has a non-homogeneous structure in which the core structure of the gel cannot be completely cross-linked due to rapid gelation. That is, while the alginate molecules on the outer surface of the alginate gel are rapidly cross-linked with Ca^2+^ ions, the alginate molecules on the inner surface of the gel may not fully participate in cross-linking. The outer surface of the gel swiftly cross-links and hardens, and the inner may remain softer, which can lead to an inhomogeneous gel structure, creating inconsistent regions in the gel structure and differences in structural integrity [41,42,43,44]. It has been stated in the literature that alginate hydrogels with slower gelation have better structural homogeneity and a higher elastic modulus than hydrogels formed during rapid gelation [44]. To cope with such a challenge, various approaches have been developed in the literature to decelerate the gel formation process, one of which is the utilization of phosphate buffers (such as sodium hexametaphosphate). Since phosphates display a higher affinity towards calcium ions than alginate molecules, the presence of phosphate ions exerts a chelating effect and delays the gelation process of alginate [38]. Another method of controlling the gelation speed is to use a different Ca^2+^ source and a controlled trigger. In this method, also known as the endogenous method, a controlled trigger [(D−glucono−δ−lactone (GDL)] and calcium source, such as calcium carbonate (CaCO_3_) or Ca-EDTA, are added to the alginate solution to ensure slow release and diffusion of Ca^2+^. In this method, a uniform gel structure is created with a slow process [39]. Kuo and Ma [42] used CaCO_3_–GDL and calcium sulphate (CaSO_4_)−CaCO_3_−GDL systems as Ca^2+^ sources and observed controlled gel formation and a more homogeneous alginate structure. In addition, it was stated in the study that the gelation rate increased as the total calcium content, CaSO_4_ ratio, and temperature increased, where the alginate concentration decreased. However, insoluble CaCO_3_ could be encapsulated into the formed gel. When the gel formation starts, the undissolved or partially dissolved CaCO_3_ particles are trapped in the gel structure. The embedding of CaCO_3_ within the gel confines the diffusion of free Ca^2+^ ions. In a model gelation process, a more uniform gel structure would occur in the presence of free ions. Contrarily, CaCO_3_ that is embedded within the gel encourages localized regions with a higher Ca^2+^ concentration, which may cause non-uniform and non-homogeneous gelation. The limited diffusion and nonuniform gelation formation affect the structural integrity and mechanical characteristics of the gel. The choice of a homogeneous Ca-EDTA solution as a calcium source to induce a controlled gelation is also another alternative approach [39].

#### 2.1.2. Barium, Ba^2+^

Ba^2+^ cations have a higher affinity towards alginate compared to Ca^2+^ cations. This is because Ba–alginate gels have higher stability properties and mechanically stronger gel characteristics than Ca–alginate gels. These gels may degrade under alkaline conditions while maintaining their stability in acidic and neutral pH environments. Ba^2+^ has affinity towards both G and M blocks, while it does not display connection propensity to MG blocks. However, the Ba^2+^ concentration is recommended to be controlled within an appropriate range when used in in vivo applications because of its toxic characteristics [39]. It must be noted that Ba^2+^ ions are used only at low concentrations in cell immobilization applications [40].

#### 2.1.3. Strontium, Sr^2+^

Sr^2+^ cations can have a strong attaching affinity towards G blocks, whereas they have limited or no affinity towards M and MG blocks. The interaction of Sr^2+^ with alginate molecular chains is explained by the core–shell structure resembling the structure of an egg-box model. Compared to Ca^2+^, Sr^2+^ has more coordination sites, which leads to stronger binding with alginate molecules. Namely, Sr–alginate gels formed at the same alginate and ion concentration have significantly higher chemical stability and stronger mechanical performance than Ca–alginate gels [39]. In contrast, Kaklamani et al. [29] detected the effect of Mg^2+^, Ca^2+^ and Sr^2+^ cations on the hydrogel properties of elasticity and gelation rate from highest to lowest as follows: Ca^2+^, Sr^2+^, and Mg^2+^. On the other hand, Ching et al. [40] stated that Sr^2+^, which is slightly toxic, should be used only at low concentrations in cell immobilization applications.

#### 2.1.4. Copper, Cu^2+^

The Cu^2+^ cation-induced gelation mechanism of alginate is quite different from that of Ca–alginate gels. In Cu–alginate gelation, four oxygen atoms (two from negatively charged carboxyl groups and the other two from neutral carboxyl groups) coordinate with a Cu^2+^ ion to form the Cu–alginate egg-box structure. The Cu^2+^ cation can interact with both G and M blocks of alginate molecular chains. The egg-box dimer, which is created between Cu^2+^ and alginate, is always longer than that created with Ca^2+^ and shows a more regular cross-linking pattern. The affinity of Cu^2+^ ions towards alginate molecular chains is ten times greater than that of the Ca^2+^ ions, which allows them to quickly transform into a dense and thick gel layer upon contact with alginate. Such a situation restricts further diffusion of Cu^2+^ throughout the gel, which makes it harder to reach the inner core. A dense and thick outer layer structure enhances the rigidity of the gel and creates a wall around it, which prevents the shrinkage of alginate gel. To induce alginate gelation for a given concentration of alginate, the amount of required Cu^2+^ is lower than that of Ca^2+^ [39]. On the other hand, as stated by Ching et al. [40], the use of Cu^2+^, which is among highly toxic cations, is limited for practical applications.

#### 2.1.5. Zinc, Zn^2+^

Although Zn^2+^ cations interact with G blocks, similar to the Ca^2+^ cation, Zn-induced gelation typically leads to mechanically weaker gel formation. Additionally, when Zn^2+^ is used in food products due to its potential side effects in terms of health, its concentration needs to be controlled within an appropriate range [39].

#### 2.1.6. Ferrous, Fe^2+^

The interaction of Fe^2+^ cations with alginate chains is similar to that of Zn^2+^ cations. Rather than M blocks, Fe^2+^ displays a higher affinity to G blocks and can allow gelation at lower Fe^2+^ levels. Having a high proportion of M blocks, alginate requires higher levels of Fe^2+^ for the formation of an integrated gel network [39]. Malektaj et al. [44] examined the mechanical properties of alginate gels cross-linked with Fe^3+^, Cu^2+^, Sr^2+^, Ca^2+^, and Zn^2+^ ions. Cross-linking with Fe^2+^ cations was found to enhance the elastic modulus of alginate gels.

#### 2.1.7. Mangane, Mn^2+^

Mn^2+^ cation can coordinate G blocks like Ca^2+^. Additionally, Mn^2+^ can also bind to M blocks via random electrostatic attraction, which leads to forming unstable complexes. Limited studies have been conducted on Mn–alginate gels as they have low affinity and form weak hydrogels [39].

### 2.2. Cross-Linking with Trivalent Cations

The binding of trivalent cations to alginate is generally stronger and more stable compared to divalent cations, resulting in a strong network structure. Divalent cations such as Ca^2+^ interact with two carboxyl groups to form a simpler network structure, while trivalent cations interact simultaneously with three carboxyl groups in different alginate macromolecules, creating a three-dimensional binding structure that results in a compact and robust gel network [40].

#### Aluminum, Al^3+^

Since the Al^3+^ cation has a three-dimensional binding structure, the stability of alginate gels created with Al^3+^ is higher than the stability of gels formed by the gels created with Ca^2+^ and Ba^2+^ cations. However, the gelation mechanism of Al–alginate is not comprehensively and clearly described in the literature. On the other hand, toxic potential of Al^3+^ restricts the use of this cation in various applications [39].

### 2.3. Cross-Linking with Chemical Bonding

Apart from ionic cross-linking, it is possible to achieve covalent cross-linking of alginate by using various chemical agents such as glutaraldehyde (GA) [37,45,46]. Because of the risk potential for bronchitis, nasal symptoms, and skin irritation in the human body and the toxic nature of GA, it is recommended to avoid using it as a cross-linker [37,47]. Alternatively, citric acid, which is considered a non-toxic chemical, has covalent cross-linker potential due to its three carboxylate groups [37]. Additionally, the use of various diamines and dihydrazides, which are used as covalent cross-linkers in hydrogel production, can also be investigated in wet-spun alginate filament production.

## 3. Principles of Textile Filament Manufacturing Methods

Wet spinning and melt spinning methods are two main common principles for converting natural-based polymers into filaments for textile materials.

### 3.1. Melt Spinning

Melt spinning technology is an effective method for processing polymers that can melt at temperatures below their decomposition temperature. Due to several challenging factors, it is not considered a suitable method for processing natural-based polymers [48,49]. For alginate, the melt spinning method is not adoptable due to its thermal sensitivity, water absorption capacity, and gelation tendency [50]. Denaturation arising from thermal sensitivity of alginate could lead to degradation of the polymer. The gelation tendency of alginate in the presence of cations may cause nozzle clogging during the process, which may further complicate the process [49,51].

### 3.2. Wet Spinning

Wet spinning technology, which for years has been employed for the manufacturing of acrylic, rayon, aramid, modacrylic, and spandex fibres, can be effectively used for the manufacturing of natural-based biodegradable filaments, provided that a suitable solvent–coagulant is determined and process parameters are optimized for the related polymer. Wet spinning is the most widely utilized spinning method for alginate in the literature. The fundamental principle of this technology relies on coagulation and solidification of the polymer due to phase inversion. After the extrusion of the polymer through a spinneret into a coagulation bath, the filaments are passed through a series of rinsing and stabilization baths and heated rollers followed by collection on a rotating spool, which is a typical practice of this method. The important parameters affecting continuous spinning and physical and mechanical properties of a natural polymer-based filament in the wet spinning process are as follows: cross-linking methods [52,53], spinning dope content and concentration, additives (plasticizers or nanoparticles) [54,55], rheological properties of the polymer solution [56], content and concentration of the coagulation bath [52], the temperature of the coagulation bath, spinneret geometry [52], the distance between the spinneret and the bath, drying and pre-drying temperature [54], drawing ratio, and post-processes such as heat treatments and stretching–drawing processes [57]. Although continuing studies exist in the literature on the processability of natural-based biodegradable polymers in the wet spinning system, alginate polymer is one of the most researched and ongoing polymers.

## 4. Mechanical Properties of Wet-Spun Alginate Filaments

Even though wet-spun alginate filaments have positive characteristics that make them appealing for specific usage areas, their scalability and widespread usage in textile applications are still restricted due to their inadequate and dubious mechanical properties. The mechanical values of wet-spun alginate filaments, such as elasticity modulus, yield strength, tensile strength, elongation, toughness, and knot strength, have pivotal importance as they determine the behavior of the filament under different load types during manufacturing and use. Accordingly, it is important to realize these terms regarding textile terminology so that the processability of the filaments through textile manufacturing technologies can be enhanced. However, we must highlight the fact that identification and modelling of the stress–strain behaviors of polymeric materials, such as textile filaments, are complicated by the diversity in their molecular structure, thermal sensitivity, time-dependent viscoelastic characteristics, anisotropic structure, and complex interactions in amorphous and crystalline regions. The mechanical parameters of wet-spun alginate filaments that were analyzed in the literature, as shown in Table 1, must be considered as guiding and leading data to produce alginate filaments with optimum characteristics for future works. It must be noted that factors including production conditions, additive use, blending with other polymers, and selected cross-linking types have significant influence on the mechanical characteristics of wet-spun alginate filaments.

### 4.1. Elasticity Modulus

Elasticity modulus (EM), which represents the attitude of a material under tension in the elastic region, is the tangent of the angle between the initial part of the stress–strain curve and the horizontal axis equal to the ratio of stress and strain in the elastic region [58]. EM determines the thresholds for classification of a material into rigid, ductile, or flexible structures [59]. In textile technologies, EM is a critical factor for the processability of a filament. While filaments with high EM deflect less, filaments with low EM, which generally have more flexibility, deflect more. EM also influences the attitude of filaments during textile manufacturing processes such as twisting, weaving, and knitting because these process techniques remarkably rely on the manipulation of filaments by the machine’s instruments. Specifically, in twisting, filaments are exposed to torsional bending forces to form a yarn. Yarns are exposed to abrupt forces in both weaving preparation and the stages of the weaving process (e.g., shedding). In the knitting process, fabrication is achieved by the way that loops are constructed with the manipulation of filaments by needles. In such a process, it is challenging to manipulate filaments with high EM because of their rigid nature. The ability of textile materials to retain their dimensional stability during use can be considered to be associated with EM. Textile fabrics with a high EM may keep their shape better, while textile fabrics with a poor EM may provide moderate flexibility but can undergo shape deformation over time. Additionally, a higher EM may result in a harsher fabric handle, while a poor EM offers a softer fabric handle [60]. The EM value of a textile material is characterized by several factors such as crystallinity degree and molecular orientation [58]. In the literature, different units including MPa, GPa, and cN/tex have been preferred by researchers in stating the EM values of alginate filaments; still, it is not possible to indicate a certain range. Conversion of units, specifically from GPa or MPa to cN/tex, is not recommended, since such a conversion would be a conceptionally contradictory issue between the material science and textile literature. The EM value range for alginate filaments, extracted from the literature, is exhibited in in detail Table 1. The test methods referenced in the studies or the test devices used are provided in the table footnote.

### 4.2. Yield Strength

Yield strength is the point at which a material starts to undergo permanent deformation and it cannot retrovert to its initial physical shape [61]. Filaments having high yield strength can keep their dimensional stability even under higher stress before undergoing permanent deformation. The stresses that filaments are exposed to during textile manufacturing processes should be limited to the yield strength of the filament. The test methods referenced in the studies or the test devices used are provided in the table footnote.

### 4.3. Tenacity

Tenacity term, which describes specific stress corresponding with peak force detected on a stress/strain curve, is recommended in textile terminology. This is because it is more practical in determining the weight of a filament per unit length. Namely, tenacity refers to strength on a gram-per-linear-density basis. In calculation, the nominal denier or tex of the filament is used as a linear density value. Scientifically, tenacity is an important criterion for analyzing the mechanical behavior of a filament, since it determines the amount of force that filament can resist and also the type of behavior under this force [62]. During textile manufacturing processes, the resistance of filaments under imparted stresses is majorly related to the tenacity of the filament. Filaments having high tenacity are less likely to rupture during processing, which promotes production efficiency and product quality [63]. When the unit of MPa is used to define the tenacity of textile filaments, a disputable issue can arise in the literature. MPa, which represents a force normalized by the area, must be preferred to express the strength of solids. In the textile literature and textile industry, specific units including gpd (gram per denier), kg/tex, and N/tex normalized by linear density are recommended for use. Such normalization in the textile literature enables an objective comparison of the tenacity of filaments with different thicknesses and densities. In the literature about alginate filaments, some researchers use the tensile strength term, whereas some prefer using the breaking strength or tenacity term. In the literature, different units including MPa, cN/dtex, cN/tex, and N have been chosen by the researchers to state the tenacity values of alginate filaments; still, it is not possible to indicate a certain range. Similar to elasticity modulus, conversion of units, specifically from MPa or N to cN/tex, is not recommended since such a conversion would be a conceptionally contradictory issue between the material science and textile literature. Tenacity-related values for alginate filaments are exhibited in Table 1 in detail. The test methods referenced in the studies or the test devices used are provided in the table footnote.

### 4.4. Knot Strength

Knot strength has a critical importance both for lateral textile manufacturing processes (such as rewinding, weaving, and knitting) and for applicable of special technical textiles, especially biomedical textiles such as surgical sutures, which serve to hold skin or other tissues after surgery or injury. The filament must be adequately resistive to hold tissues and flexible enough to make knots easily [45]. The major problem is suture breaking after knotting, which is attributed to a reduction in tensile strength resulting from knotting. It has been revealed that knot security depends on factors including the number of throws and the size and type of suture [64].

### 4.5. Elongation

Elongation indicates how much a filament can extend beyond its original size and how much it can display flexibility before rupture. For highly efficient processability in manufacturing technologies, filament elongation is of paramount importance. For example, it is reported that cotton fibers with high elongation have a high propensity to spin [65]. In the literature, the elongation values of alginate filaments, as shown in Table 1, were determined to fall in the range of 3.8–42%. The test methods referenced in the studies and the test devices used are provided in the table footnote.

### 4.6. Toughness

Toughness represents the work required to rupture a filament, which can be identifiable by the area under a stress–strain curve. Fiber toughness is derived from a combination of elongation and tensile strength [66]. In the literature, examination of the toughness values of alginate filaments has not been commonly performed by researchers.

**Table 1:** The mechanical properties of wet-spun alginate filaments.

Filament Type	Elasticity Modulus	Yield Strength	Tensile Strength	Knot Strength (cN/dtex)	Elongation (%)	Toughness (MJ·m^−3^)	Reference
Alginate ^1^	3.62 GPa		200 MPa		16		[35]
Alginate ^2^	9.01 cN/tex		17.96 cN/tex		6.63		[67]
Alginate ^3^			8.32 cN/dtex		6.97		[68]
Alginate ^4^			10.21 cN/tex		18.2		[69]
Alginate			4.3 cN/tex		20.4		[70]
Alginate ^5^			0.8–2.2 cN/dtex		5.8–20.4		[71]
Alginate ^6^			15.80 cN/tex		4.39		[72]
Alginate	3.1 gpd						[73]
Alginate ^7^			13.6 cN/tex		8		[74]
Alginate ^7^			10.2 cN/tex		5		[74]
Alginate ^7^			8.69 cN/tex		4		[74]
5% Alginate−25 G needle		116 MPa	173 MPa		18	16.16	[75]
5% Alginate−21 G needle			135 MPa		35	37.47	[75]
Alginate−10% Aluminum ^5^			20.7 cN/tex				[76]
Alginate−20% Aluminum ^5^					19.7		[76]
Alginate treated with silver nitrate ^4^			10.14 cN/tex		19.4		[69]
Alginate−Ca-DMSO	88 MPa		1.82 MPa		43		[37]
Alginate−Ba-DMSO	34 MPa		1.4 MPa		32		[37]
Alginate/chitosan ^5^			0.6- 2 cN/dtex		4.8–29.1		[71]
Alginate/chitosan ^8^	5.6–7.3 GPa	105.5–119.5 MPa	202.4–225.6 MPa		13.7–26.8		[77]
Alginate/chitosan with molecular weight 4.0 × 10^4 9^			1.0–1.5 cN/dtex	0.5			[78]
Alginate/chitosan with molecular weight 1.6 × 10^5 9^			1.0–1.6 cN/dtex	0.5–0.6			[78]
Alginate/5.11% hydrolyzed chitosan content ^10^			11.42 cN/dtex		13.60		[68]
Alginate/4.92% hydrolyzed chitosan content ^10^			4.52 cN/dtex		11.69		[68]
Alginate/4.51%−hydrolyzed chitosan content ^10^			7.88 cN/dtex		9.67		[68]
Alginate/hydrolyzed chitosan ^5^			0.7–2.7 cN/dtex		5.6–29.3		[71]
Alginate/CM−chitosan treated with HTCC ^4^			8.02–12.64 cN/tex		15.8–23.2		[79]
Alginate/CM−chitosan treated with Ag ^4^			8.12–14.50 cN/tex		14.5–21.6		[79]
Alginate/N−Succinyl-chitosan ^4^			10.32–14.32 cN/tex		20.4–43.5		[69]
Alginate/N−Succinyl-chitosan treated with silver nitrate ^4^			10.40–14.43 cN/tex		20.7–45.7		[69]
CNC/alginate (97/3) ^11^	1040.5 MPa		10.5 MPa		2.9		[80]
CNC/alginate (95/5) ^11^	769.8 MPa		11.9 MPa		6.3		[80]
CNC/alginate (90/10) ^11^	317.4 MPa		6.2 MPa		3.3		[80]
2% CNC-added alginate ^4^			2.05 cN/dtex		15.05		[56]
2–50% CNC−added alginate	2.5–6.8 gpd						[73]
MWCNT−added alginate ^2^	8.22 cN/tex		16.54 cN/tex		5.71		[67]
SWNCT−added alginate ^1^	4.01–6.97 GPa		208–250 MPa		13–18		[35]
GO−added alginate ^2^	8.50 cN/tex		20.42 cN/tex		8.71		[67]
7% Nano TiO_2_−added alginate ^6^			16.88 cN/tex		3.15		[72]
2% Nano ZnO−added alginate ^6^			18.38 cN/tex		3.62		[72]
Hydroxyapatite−added alginate ^6^			20.63–26.71 cN/tex		8.38–9.45		[81]
75/25 Gelatine/alginate ^12^					3.8		[45]
75/25 Gelatine/alginate treated with TGA enzyme ^12^			10 042 N	6.75 N	11.98		[45]
Alginate/HA by dip coating			6.13–6.58 cN/tex				[82]
Alginate/HA by dope mixing			4.20–9.059 cN/tex				[82]
Alginate/AKP ^4^			2.28–2.68 cN/dtex				[83]

^1^ ASTM D3822 Single-fiber break test. ^2^ PN EN ISO 2062:2010. ^3^ EN ISO 5079. ^4^ Using a fiber electron tensile tester. ^5^ Using a single-fiber tensile tester. ^6^ PN-EN ISO 5079:1999. ^7^ Using a yarn strength and elongation tester. ^8^ ASTM D1708-93. ^9^ ISO 2062. ^10^ EN ISO 5079. ^11^ ASTM D3822-07. ^12^ ASTM D 5034.

Studies about wet-spun alginate filaments in the literature have focused mostly the filament production processes and physical, mechanical, or thermal properties of the filaments. Most of the published research work has not extended to the lateral textile manufacturing processes of twisting, knitting, and weaving. Researchers have not yet identified the influential parameters affecting alginate filaments during the textile manufacturing phases, such as low tensile strength, poor flexibility, and low tolerance to high temperatures. To compensate for the weaknesses of wet-spun alginate filaments, unique and new research approaches to spinning process parameters and filament components need to be developed.

## 5. Process Parameters Affecting the Mechanical Properties of Wet-Spun Alginate Filaments

### 5.1. Effect of Ion Source and Alginate Spinning Dope Concentration

Ion source concentration is an important factor affecting the gelation rate of alginate and the final mechanical properties of the filament. A low ion concentration can provide flexibility to the filament, but it causes slower gelation and reduced density of cross-linking. Thus, the strength of the resultant filament may be poor. At medium ion concentration, a more balanced gelation rate and cross-linking density are achieved, which may yield optimum strength and cross-linking density. On the other hand, high ion concentration provides rapid gelation and intense cross-linking, which improve the mechanical strength of the filament, but it may lead to the production of less flexible and more brittle filaments. This also affects the continuous spinnability and processability of the filament. The concentration of calcium chloride (CaCl_2_) as a Ca^2+^ ion source has been determined to be in the range of 1–15% *w*/*v* in the literature [35,37,45,56,67,68,69,71,72,75,78,79,82,84,85,86]. Another critical factor affecting the gelation rate of alginate and the final mechanical properties of the filament is the alginate spinning dope concentration. The alginate concentration in the polymer solution, also known as the spinning dope concentration, was determined to be in the range of 0.5–7.4% *w*/*v* in the literature [35,37,45,56,67,68,69,71,72,74,75,78,79,82,84,85]. As the alginate concentration increases, the viscosity of the polymer solution also increases. Viscosity is a quite important factor that affects the processability and spinnability of the filament during the extrusion of the solution. According to literature reports, it is possible to categorize alginate concentrations into low, medium, and high viscosity ranges. Solutions with 0.5–2% concentration display lower viscosity, enabling the production of thin and flexible filaments, which allows for easier processability of the filament. In this concentration range, the tensile strength of the filament may be poor, but it offers higher flexibility and better solubility. Solutions with a concentration of 2–4% display medium viscosity, providing good processability and adequate strength. High concentration (4–6% and above) provides high cross-linking density and strength and poor flexibility [75,87,88]. Additionally, high viscosity may also bring about challenges regarding the processability and spinnability of the filament [74]. A phenomenon present in both high alginate and high Ca^2+^ concentrations is more uniform and continuous filament extrusion, which provides enhancement of the breaking strength and toughness of the resulting filament. Such an enhancement is attributed to the increased polymer content in the filament component prompting a tighter internal structure [75]. As the Ca^2+^ concentration increases, the number and size of connection zones increase. This results in higher cross-linking density and shorter binding chain segments between connecting regions. Similarly, enhancing the alginate concentration increases the cross-linking density, thus increasing the connection density, which leads to a decrease in the length of the chain segments between the connection regions. The increment in cross-linking density in the network structure leads to an increment in fracture stress. That is, the structural integrity and strength of the alginate network are directly related to the density of cross-linking [88]. LeRoux et al. [87] stated that as the concentration increases, the solid volume fraction and, accordingly, the cross-linking density increases, which leads to an increase in gel stiffness.

### 5.2. Effect of Needle Diameter

Needle diameter is a factor that directly affects the diameter of the produced filament. With smaller needle diameters, thinner filaments can be produced. It has also been proven that the needle diameter has an impact on characteristics such as filament topology, porosity, and elongation [75,89]. Chen et al. [75] examined the effect of needle diameter (21 G and 25 G) on the mechanical properties of alginate filaments and knitting fabrics. The filaments produced with a 21 G needle displayed higher elongation than those spun using a 25 G needle, indicating that a larger needle size results in higher elongation, imparting an unconsolidated structure to the filament. Additionally, it was concluded that filaments produced using a larger-diameter needle have higher toughness, with a value of 37.47 MJ*m^−3^ in a given alginate solution.

On the other hand, if the needle diameter increases, the polymer solution may spontaneously flow or drip from the needle tip due to gravity. In the combination of a large diameter and a low-viscosity solution, the possibility of solution leakage and flow under lower pressure with the influence of gravity has been stated in the literature [90]. Moreover, it is possible to correlate needle diameter and flow rate. As the needle diameter increases under constant pressure, the flow rate of the solution also increases. As the diameter increases, the area through which the fluid can pass expands, and more solution can flow simultaneously.

### 5.3. Effect of Temperature

Adjusting temperature is an alternative approach to controlling gel formation. Low temperatures allow for a reduction in the diffusion rate of ions, which brings slower cross-linking. At low temperatures, control of hydrogel formation is easier because the reactivity of the Ca^2+^ ion decreases, which prompts a more ordered network structure and thus improves mechanical characteristics [28].

### 5.4. Effect of Coagulants

Coagulants can both facilitate the diffusion of cross-linkers towards the polymer and assist in stabilizing the interaction between the polymer and cross-linker. With this motivation, some researchers have focused on the remarkable effect of varying coagulants, such as dimethyl sulfoxide (DMSO), dimethylformamide (DMF), and tetrahydrofuran (THF), on the final properties of wet-spun alginate filaments. It has been proven that Ca^2+^, Ba^2+^, and Al^3+^ filaments are more elastic when utilizing DMSO, followed by DMF and THF [37].

## 6. Different Approaches in the Literature to Producing Composite Alginate Filaments for Textile Applications

Wet-spun alginate filaments have some weaknesses, such as poor flowability during the process due to the high viscosity of the solution, poor spinnability, poor mechanical characteristics, and poor chemical properties [83,91]. The fundamental approach to improving the tenacity of a polymer-based filament is to ensure the orientation of the polymer chains by applying a stretching process to the material during production. However, the stretching process is limited in practice, and filament breaks are possible under excessive stretching force [84]. To improve the mechanical properties of filaments, such as tenacity, elasticity modulus, and toughness, or to improve their functionalities, researchers have focused on the feasibility of producing composite filaments. Some have focused on composite filaments that are structured by incorporating nanoparticles, whereas some have focused on those engineered by blending with different polymers. On the other hand, some have concentrated on the addition of grafted nanocrystal, which is a rather new approach.

### 6.1. Use of Additives

There exist many studies in the literature investigating the use of nanoparticle additions in wet-spun alginate filaments. The compatibility of the additive with the polymer, the amount of additive in solution, additive size, shape (e.g., lamellar or tubular shape), homogenous dispersion of additives, and the additive alignment are the main key parameters affecting the crystallization and mechanical characteristics of added wet-spun alginate filaments. Watthanaphanit et al. [92] observed enhancement in the stiffness of wet-spun alginate filaments in the presence of small-sized whiskers that restrain the mobility of the alginate molecules. Enhancement in mechanical properties can also be associated with alterations in the filament crystal structure via the nucleation effect of the added nanoparticles or fillers. Additives, which act as nucleating agents on the crystal phase, lead to a slight improvement in the crystallinity degree and the pseudohexagonal structure [67,72,84]. Cellulose-based nano-additives in different forms, which have attracted the most attention among additive types, are present as follows: cellulose nanocrystals (CNC) [56,73,84,93,94,95,96], and its oxidized derivative (OCNC) [96], cellulose nanofibril (CNF) [97], lignocellulose nanofibril (LCNF) [97], and TEMPO-oxidized lignocellulose nanofibril (TOLCNF) [97,98]. CNCs are widely used as fillers to enhance the properties of wet-spun alginate filaments, based on the expectation that cellulose and alginate may exhibit good compatibility. In addition, it is expected that the sulphate groups carrying negative charge of cellulose crystals may engage in an electrostatic reaction with the Ca^2+^ cations during coagulation [84]. The mechanical properties of nanoparticle or nanofiller-added wet-spun alginate filaments are influenced by the ability of alginate to transfer stress to the reinforcement, which is entirely characterized by the compatibility between the matrix and the reinforcement [99]. A challenging aspect of the addition of CNC to alginate filaments is the detriment of the orientation as the amount of CNC increases. The interaction between the polymer and the nanoparticles causes the formation of twists opposite the drawing direction, resulting in crystallites orientating themselves in a spiral pattern within the alginate polymer matrix at high concentrations. Such a spiral configuration may weaken the mechanical characteristics of filaments [100]. In the case of cellulose nanofibril use, LCNF-added alginate filaments display poor mechanical properties, because the hydrophobic characteristic of lignin may disrupt hydrogen bonding between fibrils. However, LCNF-added alginate filaments exhibited better mechanical properties than pure cellulose nanofibril-added alginate filaments. This may be attributed to a smaller diameter of LCNF due to well defibrillation during pretreatment; thus, enhanced hydrogen bonding between LCNFs brings about improved mechanical properties in LCNF alginate filaments. On the other hand, TOLCNF, which has smaller diameters than pure cellulose nanofibril, and LCNF, TOLCNF-alginate filaments show lower mechanical properties [97,101]. This may be because carboxyl groups of TOLCNF create ion bonds with Ca^2+^. Such an interaction can disrupt the “egg-box” structure and induce gel formation, leading to deteriorating mechanical properties [97]. Good interfacial interaction and compatibility between the nanocellulose and alginate have a pivotal role in enhancing the tensile strength of alginate filaments [98].

On the other hand, graphene oxide (GO) [67], multi-walled carbon nanotubes (MWCNT) [67], single-walled carbon nanotubes (SWCNT) [35], nano TiO_2_ [72,102], nano ZnO [72], silver nanoparticles [103], chitin or chitosan nanowhiskers [92,99], hydroxyapatite nano-additives [81], tricalcium phosphate nano-additives [104], and silica (SiO_2_) nano -additives [105] are among other nano-additives used to enhance the physical and mechanical characteristics of wet-spun alginate filaments. The enhancement mechanism caused by the addition of carbon nanoparticles might not solely depend on molecular alterations but perhaps on the interaction between the nanoparticles and the polymer at a supramolecular level. Although the addition of carbon nanoparticles alters the supramolecular structure of the wet-spun alginate filaments, its effect on the mechanical properties of filaments is not clearly revealed.

While nanoparticle addition has shown important potential for the enhancement of characteristics, additives at high concentrations can exhibit a propensity to agglomerate and deteriorate the homogeneity of filaments. Agglomeration results from van der Waals interactions and the large surface areas and high surface energy of the nanoparticles [68]. Agglomeration resulting from overdosed nanoparticles may also disrupt the egg-box model of the alginate [97,98]. Such nonuniform distributions lead to the weakened mechanical integrity of the filament [56,97]. In particular, exaggerated interactions between nanoparticles may cause bonded particles and nonuniform clusters, which can negatively affect mechanical properties such as the elastic modulus, tensile strength, and elongation. For this reason, the nanoparticle rate in the polymer matrix must be in the low range. Apart from mechanical characteristics, possible agglomeration of nanoparticles may cause higher filament diameters [97]. In the literature, alginate filaments with SWCNT additives reaching up to 23% by weight have been produced. In the method relying on the electrostatic interaction of SWCNT and alginate, ionic surfactant sodium dodecyl sulphate-coated nanotubes are mixed with alginate solution. However, the highest tensile strength value of 250 MPa was obtained at a 1.2 wt % concentration [35].

Another challenge involved in high amounts of added nanoparticles is the elimination of bubbles. Bubble formation affects the pumping ability of the solution through a spinneret and thus the efficient spinnability of alginate filaments. The unexpected occurrence of voids with the addition of nanoparticles may also diminish the mechanical properties of the filament due to disturbance of the egg-box structure by nanoparticles [84].

### 6.2. Preparation of Blended Filaments

Another alternative approach to producing composite filaments is combination of alginate with other polymers such as chitosan [68,70,71,77,78,79], gelatine [45], hyaluronic acid (HA) [82,85], polyvinyl alcohol [106,107], pectin [108], soy protein [86], Antarctic krill protein (AKP) [83], and CNC [80]. Blending alginate with other polymers is a widely used alginate-based membrane production technology, and it is also an approach that has attracted the attention of researchers in the wet spinning production of alginate filaments. Chitosan, which has a molecular structure similar to alginate, stands out with its biodegradable, biocompatible, antimicrobial, fungicidal, sorbent, and hemostatic properties [109]. Many researchers have investigated the wet spinnability of alginate/chitosan or modified chitosan, such as carboxymethyl chitosan (CM), N-Succinyl-chitosan (SCS), and hydrolyzed chitosan, due to advantage of a strong intermolecular interaction arising from the good polymer/polymer miscibility between the polymer molecules [69,71,77,79]. Apart from hydrogen bonding and Van der Waals Forces, a polyelectrolyte complexation (PEC) interaction occurs between polyanionic alginate and polycationic chitosan in the aqueous phase [110]. This interaction is dependent on the pH value. Alginate is only negatively charged above its p*Ka* (3.38–3.65). Chitosan with amino groups is one of the few cationic polyelectrolytes (p*Ka* ≈ 6.5) in nature [68,111]. In a suitable medium, alginate and chitosan aggregate and coagulate to create a polyelectrolyte complex [68]. In the direct blending of polymers carrying positively charged molecules (e.g., chitosan) and polymers carrying negatively charged molecules (e.g., alginate), the main challenge is the occurrence of premature gelation because of the ionic interactions of oppositely charged molecules. Such premature gelation may complicate the production of filaments from the mixture and inhibit its continuous spinnability. To cope with such a problem, chitosan can be used in emulsion form. The emulsion is prepared by adding the primary emulsion, which is obtained from olive oil and a sodium dodecyl sulphate aqueous solution, to the chitosan–citrate complex, which is formed by complexing chitosan with citric acid. The emulsified chitosan–citrate complex is added to the alginate aqueous solution, which can provide a stable mixture. At the lowest chitosan concentration (0.5% *w*/*w* chitosan), both the tensile strength and elongation of chitosan/alginate filaments are observed at the highest level [70]. According to Sweeney et al. [68], the method using emulsified chitosan may not ensure a homogeneous chitosan distribution throughout the filament length, potentially affecting the mechanical characteristics and resulting functionality of the filament. An alternative approach to producing alginate/chitosan filaments is to construct core–sheath filaments by coating the alginate filament surface with chitosan in the first coagulation bath [78]. However, the approach used in the study allowed the employment of very low chitosan rates (0.014%, 0.041% and 0.067%). This coating method was adopted by researchers in later periods and was used to combine alginate with chitosan or other polymers [71,112]. Knill et al. [71] used hydrolyzed chitosan coating in the third-step wet spinning method. In their method, it was possible to increase the incorporation level of hydrolyzed chitosan by up to 25% by weight, while the incorporation level of unhydrolyzed chitosan can range only up to 6%. This is because unhydrolyzed chitosan treatment does not give a reinforcement effect, namely, chitosan is kept in coating form instead of penetrating the filament. The hydrolysis process increases the water solubility of chitosan by lowering its molecular weight, enabling more effective adsorption of chitosan to the alginate filament. While mechanical properties are enhanced, it must be noted that high hydrolyzed chitosan content can lead to brittleness/weakness of the filaments. Sweeney et al. [68] examined the spinnability of composite filaments according to a one-step wet spinning approach in which alginate is extruded in a coagulation bath containing hydrolyzed chitosan and calcium chloride. The filaments were estimated to contain chitosan in the range of 4.50 wt % to 5.10 wt %. The production of chitosan/alginate filaments via the coating method in wet spinning technology carries the risk of non-homogeneous distribution of chitosan in the filament, causing differences in chitosan content either along filament length or between individual filaments. This may negatively affect the quality and mechanical characteristics of the filament. Sibaja et al. [77] developed chitosan-coated alginate filaments in a chitosan bath without any solution of calcium chloride.

HA is another selected polymer that is used in combination with alginate. HA, which is a linear polysaccharide-sourced polymer, is commonly utilized in the fields of controlled drug release, cellular encapsulation, tissue regeneration, and cosmetics thanks to its viscoelastic properties and biocompatibility. Additionally, HA is also quite beneficial in biomedical applications, including surgical therapies, arthritis therapy, and implants [85]. The similarity of the chemical structures of alginate and HA creates a strong interaction between the two polymers. Additionally, in the coating method, cationic calcium ions can interact with anionic HA to form calcium hyaluronate. Hussain et al. [85] produced hydrolyzed chitosan/HA-coated alginate filaments via the coating method after coagulation. In their technique, following the first coagulation bath containing CaCl_2_, alginate was passed through a hydrolyzed chitosan bath. In the final stage, the filaments were immersed in the HA bath. It was observed that the hydrolyzed chitosan and HA additive improved the mechanical properties of the filaments. This was attributed to the strong interaction between oppositely charged chitosan and HA. According to Umar et al. [82], it is difficult to achieve high-strength alginate filaments via HA coating. They investigated two different integration methods, one of which is the in situ mixture of alginate and HA and the other of which is the dip-coating method. In their study, in situ integration provided better results in terms of the mechanical characteristics of filaments. This situation occurs due to the extrusion of a homogeneous mixed polymer solution into the coagulation bath. The co-extrusion of alginate and HA increases the crystallinity of the filaments. Additionally, the linear density of HA-coated filaments increased while it decreased in the in situ mixture.

A promising approach by Zhang et al. [83] is the blending of alginate with AKP. They investigated the influence of different salt types (sodium chloride, sodium acetate, and sodium sulphate) on the mechanical properties of wet-spun AKP/alginate filaments. The highest tensile strength was observed for sodium acetate filament, wherein the types of sodium salt had a slight effect on the crystallinity. Another novel promising and inspirational approach to producing blended filament is the blending of alginate with pectin. Interactions between alginate and pectin bring about the formation of complexes enhancing biological activity through synergistic effects on cell receptors. However, this synergistic interaction between pectin and alginate is not yet completely understood. However, it is worth investigating due to its potential to be a groundbreaking development in the field of wet-spun alginate filaments [108]. Unlike studies on the use of CNC as an additive in polymers, Park et al. [80] examined the spinnability of CNC/alginate filaments in 99/1, 97/3, and 95/5 ratios. CNC with sulphate groups exhibited excellent dispersibility in polar solvents and had a strong affinity with polymers like alginate, which can serve as a binder. Such binding behavior of gelled alginate also provides good spinnability. The authors found that spinnability is greatly improved as alginate content increases in blended filaments.

### 6.3. New Approaches

The addition of grafted nanocrystal to alginate is another trending research area of recent years [93,94]. This novel technique offers improved tensile strength, flexibility, and fatigue resistance as well as being an eco-friendly approach for wet-spun alginate filaments [93,94]. In this case, CNCs have generally been selected as a template for grafting polymers. Xu et al. [93] developed CNC−covalently grafted polyaniline–alginate wet-spun composite filaments through covalent grafting of PANI onto CNCs, where Wang et al. [94] developed CNC−g−Poly (ethylene glycol) (PEG)−alginate wet-spun composite filaments. The CNC−covalently grafted polyaniline−alginate filaments were proven to exhibit stable dispersion during the wet spinning process, which makes them appropriate for industrial manufacturing. In the case of doped with CNC−g−PEG, the hydrogen bonding force between the molecules of the alginate filaments was improved, which brought about the enhanced orientation of the molecular chain as well as a greater degree of crystallinity of the composite alginate filaments. The reinforcement and toughing potential of CNC and the grafting polymer in alginate filaments was revealed. Additionally, it has been proposed that CNC’s dispersion in alginate facilitates energy absorption by serving as stress concentration zones. Grafted PEG chains have the potential to replace hydroxyl groups on the CNC surface, which leads to reduced hydrogen bonding between CNCs and alginate chains. CNC−g−PEG macromolecular chains with flexible PEG side chains augment the distance between macromolecules and enhance the mobility of molecular chains. The grafted PEG chain can enhance the adhesion between CNCs and the alginate to facilitate transfer stress along the tensile direction during the stretching process, which significantly improves the toughness of the alginate filaments. Additionally, it has been observed that the use of CNC−g−PEG with a higher molecular weight of PEG further accelerates the mobility of the alginate chains in the tensile direction [94].

## 7. Use of Different Cations in the Coagulation Phase

In addition to the studies on producing composite wet-spun alginate filaments, there is research in the literature examining the effects of the coagulation-phase conditions on the characteristics of the final filaments. Although the use of different cations in the formation of alginate hydrogel has been extensively discussed in the literature, examining their utility in the formation of textile filaments is a relatively new research field. To this end, Wang et al. [76] examined the use of a mixture of Ca^2+^ ions and Zn^2+^, Ba^2+^, Cu^2+^, and Al^3+^ cations in a coagulation bath for improving the mechanical properties of alginate filaments. The breaking strength and elongation of the filaments were observed to be improved by adding metal ions to the coagulation bath. The highest breaking strength and elongation of filaments in the coagulation bath were observed when the metal ion content was 10∼30%. When the zinc ion content was 20%, the breaking strength of the filaments increased by 46%. When the zinc ion content was 30%, the elongation value of filaments reached its maximum value. The effect of copper ion and barium ion on filaments is similar to that of zinc ion, and the maximum strength and elongation values were obtained with 20% and 30% ion content, respectively. Compared to the other three ions, aluminum ions had the strongest effect on filaments, reaching a maximum breaking strength with a value of 20.7 cN/dtex when the ion content was 10%. Aneem et al. [37] examined the effects of ionic cross-linking with different ions (Ca^2+^, Ba^2+^, and Al^3+^) on the mechanical behavior of M–alginate filaments. The strongest cross-linking was provided by the Ca^2+^ cation, followed by Ba^2+^. An interesting inspirational study for producing textile filament is research on meat analogue food technology. Cui et al. [86] investigated the effects of different salt ions (sodium chloride (NaCl)) and potassium chloride (KCl)) and their concentrations on the spinnability of alginate/soybean protein isolate (SPI) composite filaments for use in meat analogue food technology. In their study, NaCl or KCl salts were mixed with alginate/SPI in the polymer solution and extruded into a coagulation bath containing CaCl_2_. Different salt types have similar effects on the mechanical properties of filaments. It was observed that the filament strength decreased with an increase in the salt concentration. This was attributed to the addition of salt, which weakens the Ca^2+^ linking, but can additionally be attributed to the highly porous structure of the filament, which negatively affects its mechanical properties.

## 8. Functionalism of Wet-Spun Alginate Filaments

Apart from studies on improving the mechanical characteristics of wet-spun alginate filaments, there exists much research on imparting functionality to wet-spun alginate filaments. Sibaja et al. [77] investigated the biomedical applications of wet-spun alginate/chitosan filaments. The filaments showed excellent inhibition of *Escherichia coli* growth. Borkowski et al. [72] found that the addition of nano TiO_2_ and ZnO particles leads to improvement not only in mechanical characteristics but also in antibacterial functionality. Silver ion is well known for its good antibacterial properties. Fan et al. [113] converted alginate filaments into calcium/silver alginate filaments with good antibacterial activity after immersion in silver nitrate solution. Moreover, silver nanoparticles have been shown to have wound-healing properties and the ability to lower inflammatory response. Neibert et al. [103] suggested that the silver nanoparticles added to alginate filaments may improve the speed of wound healing. Xu et al. [114] developed a flame retardant and antibacterial alginate filament using the bio-based flame retardant of phytic acid and DL-arginine. On the other hand, it is possible to apply post-treatments like anti-bacterial finishing after filament spinning. Silver nitrate and N−(2−Hydroxy)−propyl−3-trimethylammonium chitosan chloride is utilized to impart antibacterial properties to alginate filaments [69,79]. To cope with the challenge of water-absorption-induced deformation of alginate filaments, Zheng et al. [115] developed superhydrophobic alginate fabrics through a dip-coating process with hexadecyltrimethoxysilane alcosol. This non-fluorinated approach, which does not require nanoparticles, leverages the hydrolysis–condensation solid product of long-chain alkyl siloxane to generate low-surface-energy roughness on the filament surface. Tian et al. [116] investigated the effect of zinc ions on the flame retardance and thermal degradation of alginate filaments employing LOI and CONE tests. It was observed that the addition of 4% zinc enhances the flame-retardant properties of the filaments. Additionally, their thermal degradation temperature increased from 210 °C to 250 °C with the addition of zinc. In a study, a flexible temperature sensor (FTS) was assembled on non-woven fabric from wet-spun alginate filaments. The produced sensor was observed to display high thermal sensitivity, a rapid response time, high accuracy, stability, and repeatability [117].

## 9. Conclusions

Although alginate, an exclusive natural-based biodegradable polymer, is widely used in film, membrane, and coating applications, its processability in textile technologies is still limited due to its inconsistent mechanical integrity, mechanical properties, and non-homogeneous structure. Firstly, to consolidate the integration and characteristics of wet-spun alginate filaments, keeping the gel formation process under control is essential. It can be concluded that the ion source’s concentration particularly affects the gelation rate and mechanical characteristics of wet-spun alginate filaments. Low ion concentrations provide flexibility but result in slower gelation and weaker cross-linking, while high concentrations provide rapid gelation, intense cross-linking, and stronger but brittle filaments. Optimal mechanical properties can be accomplished with medium ion concentrations. With the motivation of improving the mechanical characteristics of alginate filaments, nanoparticle addition, blending with other polymers, and new approaches using grafting technology have been studied in the literature. The addition of nanoparticles to alginate filaments can significantly improve mechanical properties. Small-sized whiskers and cellulose-based nanomaterials including CNC, OCNC, CNF, LCNF, and TOLCNF are especially influential in providing these improvements. However, high concentrations of nanoparticles can cause negative issues such as orientation detriment, agglomeration, and bubble formation. Other nanoparticles including GO, MWCNT, SWCNT, nano TiO_2_, nano ZnO, silver nanoparticles, chitin or chitosan nanowhiskers, hydroxyapatite, tricalcium phosphate, and SiO_2_ are also selected to enhance mechanical properties. An optimal nanoparticle concentration and good interfacial compatibility are critical factors in obtaining targeted enhancements without weakening filament structural integrity. Another method of enhancing the mechanical properties of filaments is blending alginate with other polymers such as chitosan, gelatine, HA, polyvinyl alcohol, pectin, soy protein, and AKP. Chitosan forms polyelectrolyte complexes with alginate, enhancing mechanical properties despite issues with premature gelation. HA is utilized with a dip-coating technique to improve mechanical characteristics through strong interactions with alginate. Blending with AKP and pectin also shows the potential to improve tenacity. The use of sodium acetate in AKP/alginate filaments has yielded promising results in terms of mechanical characteristics. On the other hand, the addition of grafted nanocrystals to alginate is an emerging research focus, providing enhanced tenacity and fatigue resistance. CNCs are commonly selected as templates for grafting polymers. The final filaments possess improved molecular chain orientation and crystallinity as well as enhanced toughness because of improved stress transfer along the filament. The mechanical characteristics of wet-spun alginate filaments are significantly affected by the coagulation-phase conditions and the type of cations in the coagulation bath. For instance, Zn^2+^ shows the potential to improve tenacity and elongation. Moreover, studies on functional properties such as antibacterial functionality, wound healing characteristics, flame retardancy, and water repellency to filaments have been conducted.

## 10. Future Works

The progress of wet-spun alginate filaments is promising for the textile manufacturing industry, but there are many fields in which future research can provide important insights and advances. Many studies about the mechanical properties of filaments have been conducted, yet producing wet-spun alginate filaments with optimal tenacity and breaking elongation is a challenge. Detailed investigations on the optimization of wet spinning process parameters, such as the composition and concentration of dope spinning and coagulation baths, will produce wet-spun alginate filaments with acceptable mechanical characteristics. Systematic research about these parameters’ effect on the mechanical integrity and mechanical properties of wet-spun filaments will be pivotal if we are to scale manufacturing and obtain consistent quality. Scaling up the wet spinning process of alginate filaments is still a challenge. Adaptation of alginate filament manufacturing technology to existing textile manufacturing operations is required to reveal its commercial potential. To this end, research about the different ways of enhancing mechanical characteristics, such as cross-linking techniques and coating with different polymers, can lead to substantial enhancement in filament performance. Research about the inclusion of various additives and blending alginate with other polymers will result in filaments with enhanced properties. On the other hand, using antimicrobial and conductive nanoparticles within alginate can expand the potential applications of wet-spun alginate filaments in medical textiles, smart fabrics, and sensor applications. On the other hand, studies on the biodegradability of alginate textile materials are essential if these materials are to be used sustainably in the textile industry. For instance, the testing of such materials’ biodegradability in various environmental conditions (such as soil and marine environments) would supply important findings for their life cycle assessment. Additionally, comprehensive research on the life cycle of wet-spun alginate filaments, from extraction to disposal, would offer a clear insight into their environmental impact. Finally, collaborative approaches from other disciplines including textile engineering, environmental sciences, and material science will be pivotal in manufacturing alginate filaments to meet various demands such as enhanced mechanical characteristics, acceptable biodegradability, applicability in different areas, and transition to large-scale manufacturing.

## Data Availability

No new data were created or analyzed in this study.

## References

[1] Venkatesan J., Nithya R., Sudha P.N., Kim S.-K., Kim S.K. (2014). Role of Alginate in Bone Tissue Engineering. Advances in Food and Nutrition Research.

[2] Alba K., Kontogiorgos V. (2019). Seaweed Polysaccharides (Agar, Alginate Carrageenan). Encyclopedia of Food Chemistry.

[3] Gheorghita Puscaselu R., Lobiuc A., Dimian M., Covasa M. (2020). Alginate: From Food Industry to Biomedical Applications and Management of Metabolic Disorders. Polymers.

[4] Sahoo D.R., Biswal T. (2021). Alginate and Its Application to Tissue Engineering. SN Appl. Sci..

[5] Saji S., Hebden A., Goswami P., Du C. (2022). A Brief Review on the Development of Alginate Extraction Process and Its Sustainability. Sustainability.

[6] Dudun A.A., Akoulina E.A., Zhuikov V.A., Makhina T.K., Voinova V.V., Belishev N.V., Khaydapova D.D., Shaitan K.V., Bonartseva G.A., Bonartsev A.P. (2022). Competitive Biosynthesis of Bacterial Alginate Using *Azotobacter vinelandii* 12 for Tissue Engineering Applications. Polymers.

[7] Dudun A.A., Akoulina E.A., Voinova V.V., Makhina T.K., Myshkina V.L., Zhuikov V.A., Bonartsev A.P., Bonartseva G.A. (2019). Biosynthesis of Alginate and Poly(3-Hydroxybutyrate) by the Bacterial Strain *Azotobacter agile* 12. Appl. Biochem. Microbiol..

[8] Yang J.S., Xie Y.J., He W. (2011). Research Progress on Chemical Modification of Alginate: A Review. Carbohydr. Polym..

[9] Łabowska M.B., Jankowska A.M., Michalak I., Detyna J., Kulbacka J., Biały Ł., Młynarczuk-Biały I. (2020). Applications of Alginates in the Biomedical Field. Advances in Biomedical Research—From COVID to Medical Humanities.

[10] Ahmad Raus R., Wan Nawawi W.M.F., Nasaruddin R.R. (2021). Alginate and Alginate Composites for Biomedical Applications. Asian J. Pharm. Sci..

[11] Sun L., Shen Y., Li M., Wang Q., Li R., Gong S. (2024). Preparation and Modification of Collagen/Sodium Alginate-Based Biomedical Materials and Their Characteristics. Polymers.

[12] Somogyi Škoc M., Stevelić N., Rezić I. (2024). Development and Characterization of Sustainable Coatings on Cellulose Fabric and Nonwoven for Medical Applications. Sustainability.

[13] Li Z., Wang H., Wan P., Liu Q., Xu S., Jiang J., Fan L., Tu L. (2023). Healing Evaluation of Asphalt Mixtures with Polymer Capsules Containing Rejuvenator under Different Water Solutions. Sustainability.

[14] Zhang L., Hoff I., Zhang X., Liu J., Yang C., Wang F.A. (2023). Methodological Review on Development of Crack Healing Technologies of Asphalt Pavement. Sustainability.

[15] Ribeiro T., Freire A.C., Sá-da-Costa M., Canejo J., Cordeiro V., Micaelo R. (2023). Investigating Asphalt Self-Healing with Colorless Binder and Pigmented Rejuvenator. Sustainability.

[16] Omar A., Almomani F., Qiblawey H., Rasool K. (2024). Advances in Nitrogen-Rich Wastewater Treatment: A Comprehensive Review of Modern Technologies. Sustainability.

[17] Osman M., Xiaohou S., Zhao D., Basheer A., Jin H., Zhang Y. (2019). Methane Production from Alginate-Extracted and Non-Extracted Waste of Laminaria japonica: Anaerobic Mono- and Synergetic Co-Digestion Effects on Yield. Sustainability.

[18] Sardroudi N.P., Sorolla S., Casas C., Bacardit A. (2024). A Study of the Composting Capacity of Different Kinds of Leathers, Leatherette and Alternative Materials. Sustainability.

[19] Nawaz M., Shakoor R.A., Al-Qahtani N., Bhadra J., Al-Thani N.J., Kahraman R. (2024). Polyolefin-Based Smart Self-Healing Composite Coatings Modified with Calcium Carbonate and Sodium Alginate. Polymers.

[20] Ho B.K.X., Azahari B., Yhaya M.F.B., Talebi A., Ng C.W.C., Tajarudin H.A., Ismail N. (2020). Green Technology Approach for Reinforcement of Calcium Chloride Cured Sodium Alginate Films by Isolated Bacteria from Palm Oil Mill Effluent (POME). Sustainability.

[21] El Hammadi N., Almajano M.P., Pastor M.V., Codina-Torrella I. (2024). Evaluating the Incorporation of Myrtus communis L. Leaves Infusion in Alginate-Based Films and Spheres to Enhance the Oxidative Stability of Oil-in-Water Emulsions. Polymers.

[22] Zinina O., Merenkova S., Galimov D. (2023). Development of Biodegradable Alginate-Based Films with Bioactive Properties and Optimal Structural Characteristics with Incorporation of Protein Hydrolysates. Sustainability.

[23] Kuzminova A., Dmitrenko M., Mazur A., Ermakov S., Penkova A. (2021). Novel Pervaporation Membranes Based on Biopolymer Sodium Alginate Modified by FeBTC for Isopropanol Dehydration. Sustainability.

[24] Chen J.H., Liu Q.L., Hu S.R., Ni J.C., He Y.S. (2011). Adsorption Mechanism of Cu(II) Ions from Aqueous Solution by Glutaraldehyde Crosslinked Humic Acid-Immobilized Sodium Alginate Porous Membrane Adsorbent. Chem. Eng. J..

[25] Musa M.T., Shaari N., Raduwan N.F., Kamarudin S.K., Wong W.Y. (2023). Alginate/PVA Polymer Electrolyte Membrane Modified by Hydrophilic Montmorillonite for Structure and Selectivity Enhancement for DMFC Application. Polymers.

[26] Qi M., Zhao K., Bao Q., Pan P., Zhao Y., Yang Z., Wang H., Wei J. (2019). Adsorption and Electrochemical Detection of Bovine Serum Albumin Imprinted Calcium Alginate Hydrogel Membrane. Polymers.

[27] https://www.scopus.com/results/results.uri?sort=plf-f&src=s&st1=alginate+wet+spinning+filament&sid=dac5028265ca65fb879c0bf9328a3928&sot=b&sdt=b&sl=45&s=ALL%28alginate+AND+wet+AND+spinning+AND+filament%29&origin=searchbasic&editSaveSearch=&yearFrom=Before+1960&yearTo=Present&sessionSearchId=dac5028265ca65fb879c0bf9328a3928&limit=10.

[28] Augst A.D., Kong H.J., Mooney D.J. (2006). Alginate Hydrogels as Biomaterials. Macromol. Biosci..

[29] Kaklamani G., Cheneler D., Grover L.M., Adams M.J., Bowen J. (2014). Mechanical Properties of Alginate Hydrogels Manufactured Using External Gelation. J. Mech. Behav. Biomed. Mater..

[30] Zhang X., Wang X., Fan W., Liu Y., Wang Q., Weng L. (2022). Fabrication, Property and Application of Calcium Alginate Fiber: A Review. Polymers.

[31] Templeman J.R., Rogers M.A., Cant J.P., McBride B.W., Osborne V.R. (2018). Effects of a Wax Organogel and Alginate Gel Complex on Holy Basil (*Ocimum sanctum*) In Vitro Ruminal Dry Matter Disappearance and Gas Production. J. Sci. Food Agric..

[32] Topuz F., Henke A., Richtering W., Groll J. (2012). Magnesium Ions and Alginate Do Form Hydrogels: A Rheological Study. Soft Matter.

[33] Lee K.Y., Mooney D.J. (2012). Alginate: Properties and Biomedical Applications. Prog. Polym. Sci..

[34] Draget K.I., Taylor C. (2011). Chemical, Physical and Biological Properties of Alginates and Their Biomedical Implications. Food Hydrocoll..

[35] Sa V., Kornev K.G.A. (2011). Method for Wet Spinning of Alginate Fibers with a High Concentration of Single-Walled Carbon Nanotubes. Carbon.

[36] Qin Y. (2008). Alginate Fibres: An Overview of the Production Processes and Applications in Wound Management. Polym. Int..

[37] Aneem T.H., Wong S.Y., Afrin H., Nurunnabi M., Li X., Arafat M.T. (2021). Investigation of Coagulation Process of Wet-Spun Sodium Alginate Polymannuronate Fibers with Varied Functionality Using Organic Coagulants and Cross-Linkers. Mater. Today Chem..

[38] Cattelan G., Guerrero Gerbolés A., Foresti R., Pramstaller P.P., Rossini A., Miragoli M., Caffarra Malvezzi C. (2020). Alginate Formulations: Current Developments in the Race for Hydrogel-Based Cardiac Regeneration. Front. Bioeng. Biotechnol..

[39] Hu C., Lu W., Mata A., Nishinari K., Fang Y. (2021). Ions-Induced Gelation of Alginate: Mechanisms and Applications. Int. J. Biol. Macromol..

[40] Ching S.H., Bansal N., Bhandari B. (2017). Alginate Gel Particles–A Review of Production Techniques and Physical Properties. Crit. Rev. Food Sci. Nutr..

[41] Drury J.L., Dennis R.G., Mooney D.J. (2004). The Tensile Properties of Alginate Hydrogels. Biomaterials.

[42] Kuo C.K., Ma P.X. (2001). Ionically Crosslinked Alginate Hydrogels as Scaffolds for Tissue Engineering: Part 1. Structure, Gelation Rate and Mechanical Properties. Biomaterials.

[43] Skjåk-Bræk G., Grasdalen H., Smidsrød O. (1989). Inhomogeneous Polysaccharide Ionic Gels. Carbohydr. Polym..

[44] Malektaj H., Drozdov A.D., deClaville Christiansen J. (2023). Mechanical Properties of Alginate Hydrogels Cross-Linked with Multivalent Cations. Polymers.

[45] Eriningsih R., Marlina R. (2014). Pre-Clinical Research of Gelatin/Alginate Yarn for Medical Textile. Sci. Res. J..

[46] Kim Y.J., Yoon K.J., Ko S.W. (2000). Preparation and Properties of Alginate Superabsorbent Filament Fibers Crosslinked with Glutaraldehyde. J. Appl. Polym. Sci..

[47] Takigawa T., Endo Y. (2006). Effects of Glutaraldehyde Exposure on Human Health. J. Occup. Health.

[48] Hufenus R., Yan Y., Dauner M., Kikutani T. (2020). Melt-Spun Fibers for Textile Applications. Materials.

[49] Var C., Palamutcu S., Muthu S.S. (2024). Man-Made Bio-Based and Biodegradable Fibers for Textile Applications. Sustainable Manufacturing Practices in the Textiles and Fashion Sector. Sustainable Textiles: Production, Processing, Manufacturing & Chemistry.

[50] Zdiri K., Cayla A., Elamri A., Erard A., Salaun F. (2022). Alginate-Based Bio-Composites and Their Potential Applications. J. Funct. Biomater..

[51] Hu C., Lu W., Sun C., Zhao Y., Zhang Y., Fang Y. (2022). Gelation Behavior and Mechanism of Alginate with Calcium: Dependence on Monovalent Counterions. Carbohydr. Polym..

[52] Meyer M., Baltzer H., Schwikal K. (2010). Collagen Fibres by Thermoplastic and Wet Spinning. Mater. Sci. Eng. C.

[53] Tonndorf R., Gossla E., Aibibu D., Lindner M., Gelinsky M., Cherif C. (2018). Wet Spinning and Riboflavin Crosslinking of Collagen Type I/III Filaments. Biomed. Mater..

[54] Kim H.C., Kim D., Lee J.Y., Zhai L., Kim J. (2019). Effect of Wet Spinning and Stretching to Enhance Mechanical Properties of Cellulose Nanofiber Filament. Int. J. Precis. Eng. Manuf. Technol..

[55] Yue C., Ding C., Du X., Cheng B. (2022). Novel Collagen/GO-MWNT Hybrid Fibers with Improved Strength and Toughness by Dry-Jet Wet Spinning. Compos. Interfaces.

[56] Liu J., Zhang R., Ci M., Sui S., Zhu P. (2019). Sodium Alginate/Cellulose Nanocrystal Fibers with Enhanced Mechanical Strength Prepared by Wet Spinning. J. Eng. Fiber. Fabr..

[57] Al Faruque M.A., Remadevi R., Razal J.M., Naebe M. (2020). Impact of the Wet Spinning Parameters on the Alpaca-Based Polyacrylonitrile Composite Fibers: Morphology and Enhanced Mechanical Properties Study. J. Appl. Polym. Sci..

[58] Lal Regar M., Ram Meena C., Singh Hada J., Chattopadhyay R., Sinha S.K., Regar L. (2023). Fiber Testing. Textile Calculation Fibre to Finished Garment.

[59] Farag R., Elmogahzy Y., Bunsell A.R. (2018). Tensile Properties of Cotton Fibers. Handbook of Properties of Textile and Technical Fibres.

[60] Wang X., Liu X., Deakin C.H. (2008). Physical and Mechanical Testing of Textiles. Fabric Testing.

[61] Jafferson J.M., Chatterjee D. (2021). A Review on Polymeric Materials in Additive Manufacturing. Mater. Today Proc..

[62] Savile B.P. (1999). Physical Testing of Textiles.

[63] Meredith R. (1952). Properties of Textile Materials. I—Tensile Strength, Breaking Extension and Stress-Strain Relations of Fibres. J. Text. Inst. Proc..

[64] Kim J.C., Lee Y.K., Lim B.S., Rhee S.H., Yang H.C. (2007). Comparison of Tensile and Knot Security Properties of Surgical Sutures. J. Mater. Sci. Mater. Med..

[65] Mathangadeera R.W., Hequet E.F., Kelly B., Dever J.K., Kelly C.M. (2020). Importance of Cotton Fiber Elongation in Fiber Processing. Ind. Crops Prod..

[66] Moriam K., Sawada D., Nieminen K., Hummel M., Ma Y., Rissanen M., Sixta H. (2021). Towards Regenerated Cellulose Fibers with High Toughness. Cellulose.

[67] Szparaga G., Brzezińska M., Pabjańczyk-Wlazło E., Puchalski M., Sztajnowski S., Krucińska I. (2020). Structure–Property of Wet-Spun Alginate-Based Precursor Fibers Modified with Nanocarbons. Autex Res. J..

[68] Sweeney I.R., Miraftab M., Collyer G. (2014). Absorbent Alginate Fibres Modified with Hydrolysed Chitosan for Wound Care Dressings—II. Pilot Scale Development. Carbohydr. Polym..

[69] Fan L., Yu L., Xu Y., Yi C., Cai J., Li M., Huang J. (2010). The Novel Alginate/N-Succinyl-Chitosan Antibacterial Blend Fibers. J. Appl. Polym. Sci..

[70] Watthanaphanit A., Supaphol P., Furuike T., Tokura S., Tamura H., Rujiravanit R. (2009). Novel Chitosan-Spotted Alginate Fibers from Wet-Spinning of Alginate Solutions Containing Emulsified Chitosan−Citrate Complex and Their Characterization. Biomacromolecules.

[71] Knill C.J., Kennedy J.F., Mistry J., Miraftab M., Smart G., Groocock M.R., Williams H.J. (2004). Alginate Fibres Modified with Unhydrolysed and Hydrolysed Chitosans for Wound Dressings. Carbohydr. Polym..

[72] Borkowski D., Krucińska I., Draczyński Z. (2020). Preparation of Nanocomposite Alginate Fibers Modified with Titanium Dioxide and Zinc Oxide. Polymers.

[73] Ureña-Benavides E.E., Kitchens C.L. (2011). Wide-Angle X-ray Diffraction of Cellulose Nanocrystal−Alginate Nanocomposite Fibers. Macromolecules.

[74] Xu G.K., Liu L., Yao J.M. (2013). Fabrication and Characterization of Alginate Fibers by Wet-Spinning. Adv. Mater. Res..

[75] Chen Z., Song J., Xia Y., Jiang Y., Murillo L.L., Tsigkou O., Wang T., Li Y. (2021). High Strength and Strain Alginate Fibers by a Novel Wheel Spinning Technique for Knitting Stretchable and Biocompatible Wound-Care Materials. Mater. Sci. Eng. C.

[76] Wang Q., Zhang L., Liu Y., Zhang G., Zhu P. (2020). Characterization and Functional Assessment of Alginate Fibers Prepared by Metal-Calcium Ion Complex Coagulation Bath. Carbohydr. Polym..

[77] Sibaja B., Culbertson E., Marshall P., Boy R., Broughton R.M., Solano A.A., Esquivel M., Parker J., De La Fuente L., Auad M.L. (2015). Preparation of Alginate–Chitosan Fibers with Potential Biomedical Applications. Carbohydr. Polym..

[78] Tamura H., Tsuruta Y., Tokura S. (2002). Preparation of Chitosan-Coated Alginate Filament. Mater. Sci. Eng. C.

[79] Fan L., Du Y., Zhang B., Yang J., Zhou J., Kennedy J.F. (2006). Preparation and Properties of Alginate/Carboxymethyl Chitosan Blend Fibers. Carbohydr. Polym..

[80] Park J.S., Park C.W., Han S.Y., Lee E.A., Azelia Wulan C., Kim J.K., Kwon G.J., Seo Y.H., Youe W.J., Gwon J. (2021). Preparation and Properties of Wet-Spun Microcomposite Filaments from Cellulose Nanocrystals and Alginate Using a Microfluidic Device. BioResources.

[81] Boguń M., Mikołajczyk T., Rabiej S. (2009). Effect of Formation Conditions on the Structure and Properties of Nanocomposite Alginate Fibers. J. Appl. Polym. Sci..

[82] Umar M., Ullah A., Nawaz H., Areeb T., Hashmi M., Kharaghani D., Kim K.O., Kim I.S. (2021). Wet-Spun Bi-Component Alginate Based Hydrogel Fibers: Development and In-Vitro Evaluation as a Potential Moist Wound Care Dressing. Int. J. Biol. Macromol..

[83] Zhang R., Guo J., Liu Y., Chen S., Zhang S., Yu Y. (2018). Effects of Sodium Salt Types on the Intermolecular Interaction of Sodium Alginate/Antarctic Krill Protein Composite Fibers. Carbohydr. Polym..

[84] Ureña-Benavides E.E., Brown P.J., Kitchens C.L. (2010). Effect of Jet Stretch and Particle Load on Cellulose Nanocrystal−Alginate Nanocomposite Fibers. Langmuir.

[85] Hussain T., Masood R., Umar M., Areeb T., Ullah A. (2016). Development and Characterization of Alginate-Chitosan-Hyaluronic Acid (ACH) Composite Fibers for Medical Applications. Fibers Polym..

[86] Cui B., Mao Y., Liu J., Liang X., Wu D., Chen X., Wang X., Liang H., Li J., Zhou B. (2023). Effect of Salt on Solution Behavior of Spinning Medium and Properties of Meat Analogue Fibers. Food Hydrocoll..

[87] LeRoux M.A., Guilak F., Setton L.A. (1999). Compressive and Shear Properties of Alginate Gel: Effects of Sodium Ions and Alginate Concentration. J. Biomed. Mater. Res..

[88] Zhang J., Daubert C.R., Foegeding E.A. (2005). Fracture Analysis of Alginate Gels. J. Food Sci..

[89] Zhou T., NajafiKhoshnoo S., Esfandyarpour R., Kulinsky L. (2023). Dissolvable Calcium Alginate Microfibers Produced via Immersed Microfluidic Spinning. Micromachines.

[90] Lin H.Y., Wang H.W. (2012). The Influence of Operating Parameters on the Drug Release and Antibacterial Performances of Alginate Fibrous Dressings Prepared by Wet Spinning. Biomatter.

[91] Sang Z., Zhang W., Zhou Z., Fu H., Tan Y., Sui K., Xia Y. (2017). Functionalized Alginate with Liquid-Like Behaviors and Its Application in Wet-Spinning. Carbohydr. Polym..

[92] Watthanaphanit A., Supaphol P., Tamura H., Tokura S., Rujiravanit R. (2010). Wet-Spun Alginate/Chitosan Whiskers Nanocomposite Fibers: Preparation, Characterization and Release Characteristic of the Whiskers. Carbohydr. Polym..

[93] Xu Z., Zhou J., Li D., Zhu G., Lin N. (2023). Flexible Conductive Fibers from Alginate, Cellulose Nanocrystals, and Polyaniline by Wet Spinning. ACS Sustain. Chem. Eng..

[94] Wang Y., Chen H., Cui L., Tu C., Yan C., Guo Y. (2022). Toughen and Strengthen Alginate Fiber by Incorporation of Polyethylene Glycol Grafted Cellulose Nanocrystals. Cellulose.

[95] Ureña-Benavides E.E., Kitchens C.L. (2012). Cellulose Nanocrystal Reinforced Alginate Fibers—Biomimicry Meets Polymer Processing. Mol. Cryst. Liq. Cryst..

[96] Ma X., Li R., Zhao X., Ji Q., Xing Y., Sunarso J., Xia Y. (2017). Biopolymer Composite Fibres Composed of Calcium Alginate Reinforced with Nanocrystalline Cellulose. Compos. Part A Appl. Sci. Manuf..

[97] Park J.S., Park C.W., Han S.Y., Lee E.A., Cindradewi A.W., Kim J.K., Kwon G.J., Seo Y.H., Yoo W.J., Gwon J. (2021). Preparation and Properties of Wet-Spun Microcomposite Filaments from Various CNFs and Alginate. Polymers.

[98] Shen X.J., Huang P.L., Chen J.H., Wu Y.Y., Liu Q.Y., Sun R.C. (2017). Comparison of Acid-Hydrolyzed and TEMPO-Oxidized Nanocellulose for Reinforcing Alginate Fibers. BioResources.

[99] Watthanaphanit A., Supaphol P., Tamura H., Tokura S., Rujiravanit R. (2008). Fabrication, Structure, and Properties of Chitin Whisker-Reinforced Alginate Nanocomposite Fibers. J. Appl. Polym. Sci..

[100] Reddy N., Yang Y. (2015). Innovative Biofibers from Renewable Resources.

[101] Ren N., Qiao A., Cui M., Huang R., Qi W., Su R. (2023). Design and Fabrication of Nanocellulose-Based Microfibers by Wet Spinning. Chem. Eng. Sci..

[102] Papageorgiou S.K., Katsaros F.K., Favvas E.P., Romanos G.E., Athanasekou C.P., Beltsios K.G., Tzialla O.I., Falaras P. (2012). Alginate Fibers as Photocatalyst Immobilizing Agents Applied in Hybrid Photocatalytic/Ultrafiltration Water Treatment Processes. Water Res..

[103] Neibert K., Gopishetty V., Grigoryev A., Tokarev I., Al-Hajaj N., Vorstenbosch J., Philip A., Minko S., Maysinger D. (2012). Wound-Healing with Mechanically Robust and Biodegradable Hydrogel Fibers Loaded with Silver Nanoparticles. Adv. Healthc. Mater..

[104] Mikołajczyk T., Boguń M., Kurzak A., Szparaga G. (2009). Zinc Alginate Fibres with a Tricalcium Phosphate (TCP) Nanoadditive. FIBRES Text. East. Eur..

[105] Mikołajczyk T., Boguń M., Rabiej S., Król P. (2010). Zinc Alginate Fibres with a Silica (SiO2) Nanoadditive. FIBRES Text. East. Eur..

[106] Fahma F., Febiyanti I., Lisdayana N., Sari Y.W., Noviana D., Yunus M., Kadja G.T.M., Kusumaatmaja A. (2022). Production of Polyvinyl Alcohol–Alginate–Nanocellulose Fibers. Starch Stärke.

[107] Fan L., Du Y., Wang X., Huang R., Zhang L., Hu L. (2005). Preparation and Characterization of Alginate/Poly(Vinyl Alcohol) Blend Fibers. J. Macromol. Sci. Part A.

[108] Davydova G.A., Chaikov L.L., Melnik N.N., Gainutdinov R.V., Selezneva I.I., Perevedentseva E.V., Mahamadiev M.T., Proskurin V.A., Yakovsky D.S., Mohan A.G. (2024). Polysaccharide Composite Alginate–Pectin Hydrogels as a Basis for Developing Wound Healing Materials. Polymers.

[109] Zhuikova Y.V., Zhuikov V.A., Khaydapova D.D., Lunkov A.P., Bonartseva G.A., Varlamov V.P. (2024). Evaluation of Chemical and Biological Properties of Biodegradable Composites Based on Poly(3-hydroxybutyrate) and Chitosan. Polymers.

[110] Azevedo F.F., Cantarutti T.A., Remiro P.D.F.R., Barbieri B., Azoubel R.A., Nagahara M.H.T., Moraes A.M., Lima M.H.M. (2022). Histological and Molecular Evidence of the Positive Performance of Glycerol-Plasticized Chitosan-Alginate Membranes on Skin Lesions of Hyperglycemic Mice. Polymers.

[111] Zhuikova Y., Zhuikov V., Varlamov V. (2022). Biocomposite Materials Based on Poly(3-hydroxybutyrate) and Chitosan: A Review. Polymers.

[112] Tamura H., Tsuruta Y., Itoyama K., Worakitkanchanakul W., Rujiravanit R., Tokura S. (2004). Preparation of Chitosan Filament Applying New Coagulation System. Carbohydr. Polym..

[113] Fan L., Zhu H., Zheng H., Xu Y., Zhang C. (2007). Structure and Properties of Blend Fibers Prepared from Alginate and Konjac Glucomannan. J. Appl. Polym. Sci..

[114] Xu J., Jiang Z., Hou F., Zhu K., Xu C., Wang C., Wang H. (2022). Preparation and Mechanism of Bio-Based Sodium Alginate Fibers with Flame Retardant and Antibacterial Properties. Polymers.

[115] Zheng C., Sun Y., Cui Y., Yang W., Lu Z., Shen S., Xia Y., Xiong Z. (2021). Superhydrophobic and Flame-Retardant Alginate Fabrics Prepared through a One-Step Dip-Coating Surface-Treatment. Cellulose.

[116] Tian G., Ji Q., Xu D., Tan L., Quan F., Xia Y. (2013). The Effect of Zinc Ion Content on Flame Retardance and Thermal Degradation of Alginate Fibers. Fibers Polym..

[117] Wang F., Jiang J., Sun F., Sun L., Wang T., Liu Y., Li M. (2020). Flexible Wearable Graphene/Alginate Composite Non-Woven Fabric Temperature Sensor with High Sensitivity and Anti-Interference. Cellulose.

